# ﻿Taxonomic update of *Pycnoscelus* Scudder, 1862 (Blattodea, Blaberidae, Pycnoscelinae), with descriptions of two new species from China

**DOI:** 10.3897/zookeys.1231.141287

**Published:** 2025-03-10

**Authors:** Yi-Shu Wang, Xiao-Jiao Dong, Zong-Qing Wang, Yan-Li Che

**Affiliations:** 1 Yibin Academy of Southwest University, Yibin, Sichuan 644000, China; 2 College of Plant Protection, Southwest University, Beibei, Chongqing 400715, China; 3 Key Laboratory of Agricultural Biosafety and Green Production of Upper Yangtze River (Ministry of Education), Southwest University, Chongqing 400715, China

**Keywords:** China, cockroach, new species, *Pycnoscelus*, species group

## Abstract

In this paper knowledge of the genus *Pycnoscelus* Scudder, 1862 is updated. Two new species from southern China are described: *Pycnoscelusputeus* Wang & Che, **sp. nov.** and *P.undulatus* Wang & Che, **sp. nov.** Diagnostic characters and high-definition morphological photographs are provided for five known species and two species unidentified due to limited information are reported. An updated checklist and key for *Pycnoscelus* species worldwide are provided.

## ﻿Introduction

The cockroach genus *Pycnoscelus* (Blattodea, Blaberidae, Pycnoscelinae) currently comprises 16 recognized species, most of which are distributed in the Oriental Region (Roth 1998; Anisyutkin 2002, 2018). Species of this genus are remarkable within Blaberidae for their shortened fore tibiae with Type C_1_ front femur, the distinct asymmetrical subgenital plate with a relatively large right style, and a small or absent left style. Additionally, the dorsal surface of the nymph, from the fourth abdominal tergum to the supra-anal plate, is clearly rough, adorned with small tubercles (Wang et al. 2024).

The type species *Pycnoscelussurinamensis* (Linnaeus, 1758) is a widely distributed pest and notable for being exclusively parthenogenetic (Roth and Willis 1956; Roth 1974). Prior to 1967, *Pycnoscelusindicus* (Fabricius, 1775) was considered the bisexual form of *P.surinamensis*. After a series of biological experiments, Roth (1967) eventually confirmed that the bisexual relatives of *P.surinamensis* represent a separate species, *P.indicus*. Furthermore, Roth (1973, 1998) examined most type specimens in the genus, providing detailed descriptions of 12 recognized species, which serve as essential references for future taxonomic studies on *Pycnoscelus*. Since 2000, the documentation of *Pycnoscelus* species has been updated primarily by Anisyutkin (2002, 2004, 2018), who described four new species and redescribed two known ones. Nonetheless, a revision of *Pycnoscelus* remains necessary because the species described in Roth (1998) lack detailed descriptions of male genitalia, and the drawn illustrations provided were inadequate.

In this paper, we aim to advance the taxonomic knowledge of *Pycnoscelus*, providing descriptions of two new species and additional details for five known species. We also report two unidentified species. High-definition images and diagnoses of these *Pycnoscelus* species are provided, with an updated checklist and key for the genus.

## ﻿Materials and methods

All specimens examined in this paper, including the types of new species, are deposited in College of Plant Protection, Southwest University, Chongqing, China (**SWU**). Dissections of male genitalia followed the protocols described in Wang et al. (2021). Photographs were taken with a Leica DFC digital microscope camera attached to a Leica M205A stereomicroscope, and all images were processed in Adobe Photoshop CS6. Descriptions of new species are based on the holotype male, with measurement ranges provided for all type material. Terminology for male genitalia sclerites follows Klass (1997) and Anisyutkin (2018).

## ﻿Taxonomic account

### ﻿Genus Pycnoscelus

Taxon classificationAnimaliaBlattodeaBlaberidae

Scudder, 1862

2BCA4C78-EFCA-5052-B6BC-B76A4512BE00

Fig. 1A–C

Pycnoscelus Scudder, 1862: 421; Hebard 1917: 269; Roth 1998: 94; Anisyutkin 2018: 80. Type species: Pycnoscelussurinamensis (Linnaeus, 1758) = Pycnoscelusobscurus Scudder, 1862, nymph, adventive, by monotypy.
Epilampria Tepper, 1894: 174. Type species: Pycnoscelussurinamensis (Linnaeus, 1758) = Epilampriatatei Tepper, by monotypy. Synonymized by Princis 1964: 263.

#### Diagnosis.

**Figure 1.**
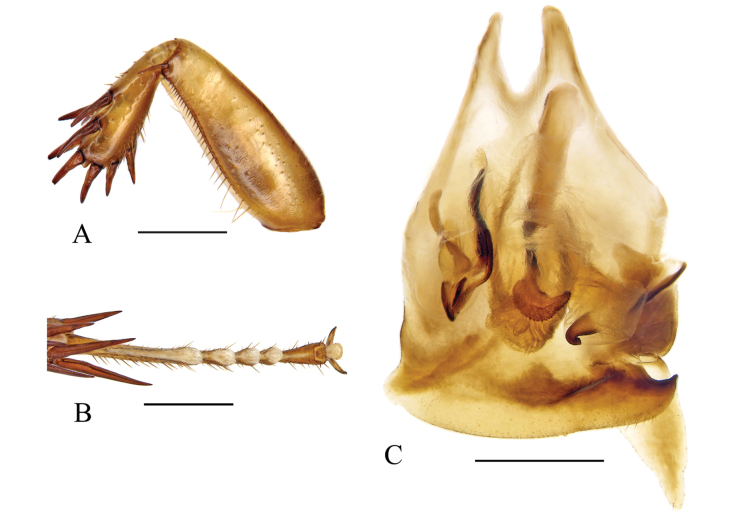
Diagnostic features of *Pycnoscelus* (*P.indicus*) **A** front femur and tibiae, ventral view **B** hind tarsi and pulvilli, ventral view **C** male genitalia and subgenital plate, dorsal view. Scale bars: 1.0 mm (**A–C**).

*Pycnoscelus* species can be easily recognized by the shortened fore tibiae, which is thickened distally with strong spines (Fig. 1A), as well as the right posterolateral corner of male subgenital plate angularly produced and upturned (Fig. 1C).

#### Description.

Facial part of head and pronotum with distinct punctations. Pronotum with the anterior margin nearly truncate, hind margin convex medially. Anterior margin of fore femur Type C_1_. Hind metatarsus longer or nearly equal to the remaining segments combined; armed with large pulvilli, occupying more than half length of the segment (Fig. 1B). Claws symmetrical and unspecialized, arolium present. Subgenital plate covered by the eighth sternum; right style typically large. Male genitalia: right phallomere with R3 forked caudally, and the left branch usually wider than the right; R5 sometimes absent. Apical part of sclerite L2D well sclerotized, with the sac-like apical membrane wrapped. Sclerite L3 hooked with small, curved portion. Sclerite L4U distinct and divided into two parts.

##### ﻿Worldwide checklist and species grouping of *Pycnoscelus*

Two groups of *Pycnoscelus* species were established based on the shape of right style (Roth 1998):

1. *indicus* species group: the right style elongate with apex directed posteriorly; includes 12 species: *P.conferta* (Walker, 1869: 148); *P.femapterus* Roth, 1998: 108; *P.gorochovi* Anisyutkin, 2002: 352; *P.indicus* (Fabricius, 1775: 272); *P.janetscheki* Bey-Bienko, 1968: 60; *P.nigra* (Brunner von Wattenwyl, 1865: 280); *P.puteus* sp. nov.; *P.rothi* Anisyutkin, 2002: 355; *P.schwendingeri* Anisyutkin, 2018: 80; *P.surinamensis* (Linnaeus, 1758: 424); *P.undulatus* sp. nov.; *P.vietnamensis* Anisyutkin, 2002: 355.
2. *striatus* species group: the right style broader, plate-like, with apex directed to the left; includes five species: *P.aurantius* Hanitsch, 1935: 18; *P.rufus* Bey-Bienko, 1950: 268; *P.semivitreus* Princis, 1967: 148; *P.striatus* (Kirby, 1903: 378); *P.tenebrigera* (Walker, 1868: 31).

Note: *P.micropterus* Hanitsch, 1931 has not been attributed to any group due to the lack of information on its right style. Additionally, there are six unnamed species: *Pycnoscelus* sp. A Roth, 1998: 121; *Pycnoscelus* sp. B Roth, 1998: 123; *Pycnoscelus* sp. C Roth, 1998: 123; *Pycnoscelus* sp. D Lucañas & Lit, 2016: 9; *Pycnoscelus* sp. E (Malaysia, Borneo); *Pycnoscelus* sp. F (China, Yunnan). The latter two species are included in this work.

### ﻿Key to males of the genus *Pycnoscelus* (updated from Roth 1998)

**Table:** 

1	Tegmina reaching only to the second tergum	***P.micropterus* Hanitsch, 1931**
–	Tegmina not as above	**2**
2	Right style elongate and cone-shaped	**3**
–	Right style broad and plate-like	**13**
3	Pronotum dark with a broad anterior and narrow anterolateral yellowish band	***P.indicus* (Fabricius, 1775) and *P.surinamensis* (Linnaeus, 1758)**
–	Pronotum not as above	**4**
4	Pronotum with large pale areas, and the disk with large blackish macula	**5**
–	Pronotum with or without small pale areas anteriorly and anterolaterally	**7**
5	The large pale areas of pronotum scattered with dark dots	**6**
–	The large pale areas of pronotum without scattered dark dots	***P.conferta* (Walker, 1869)**
6	The macula on pronotal disk irregular	***P.schwendingeri* Anisyutkin, 2018**
–	The macula on pronotal disk regular and complete	***P.rothi* Anisyutkin, 2002**
7	Supra-anal plate with hind margin deeply concave at the right side	**8**
–	Supra-anal plate not as above	**10**
8	Head and pronotum mostly black	**9**
–	Head and pronotum reddish brown; right style more bulky	***P.undulatus* sp. nov.**
9	Subgenital plate with distinct projection on left posterolateral angle	***P.gorochovi* Anisyutkin, 2002**
–	Subgenital plate without distinct projection on left posterolateral angle	***P.vietnamensis* Anisyutkin, 2002**
10	General color shiny, black or dark brown	**11**
–	Pronotum mostly black, tegmina yellowish brown with anal field and proximal region of the posterior field darker	***P.femapterus* Roth, 1998**
11	Interocular space nearly equal to or narrower than the distance between ocellar spots, hind margin of supra-anal plate with a shallow medial excavation	***P.nigra* (Brunner, 1865)**
–	Interocular space wider than the distance between inner margins of ocellar spots	**12**
12	Pronotum with moderately broad yellow areas anterolaterally, tegmina and wings extending beyond the apex of the abdomen by just over half the length of the pronotum	***P.janetscheki* Bey-Bienko, 1968**
–	Pronotum with two indistinct and narrow yellow borders anterolaterally, tegmina and wings extending well beyond end of abdomen	***P.puteus* sp. nov.**
13	Right style huge, extends to the left posterolateral angle of subgenital plate	***P.semivitreus* Princis, 1967**
–	Right style not as above	**14**
14	Tegmina and wings reduced, reaching to approximately the seventh tergum	***P.striatus* (Kirby, 1903)**
–	Tegmina and wings well developed, extending beyond end of abdomen	**15**
15	General color yellowish brown; tegmina bicolored with proximal parts reddish brown; right style as in Anisyutkin (2004: fig. 5)	***P.rufus* Bey-Bienko, 1950**
–	General color darker, reddish brown or orangish	**16**
16	Tegmina distinctly bicolored, with proximal parts reddish brown and remaining part pale yellowish (Fig. 8G)	***P.striatus* (Kirby, 1903)**
–	Tegmina coloration not as above	**17**
17	Right style relatively small, with the apex directed to the left and rounded (Fig. 7G)	***P.aurantius* Hanitsch, 1935**
–	Right style relatively large, with the apex directed to the left and sharp, as in Roth (1998: fig. 37, 38)	***P.tenebrigera* (Walker, 1868)**

### ﻿ Pycnoscelus indicus

Taxon classificationAnimaliaBlattodeaBlaberidae

(Fabricius, 1775)

72F47432-B1A0-54F5-9089-7242613C07D4

Fig. 2

Blatta indica Fabricius, 1775: 272.
Pycnoscelus indicus : Roth 1967: 774; Roth 1998: 99.

#### Material examined.

China • 5 males & 3 females; Fujian Prov., Putian City, Meizhou Island; 22–23 July 2013; Shun-Hua Gui & Yan Shi leg. • 2 males; Guangdong Prov., Zhongshan City, Zimaling Park, 11 Oct. 2018; Ke-Liang Wu leg. • 1 female; Guangxi Prov., Baise City, Renzhuang Township, Tengmao Village, Nongli tun; 9–13 July 2015; Jian-Yue Qiu leg. • 1 male; Guangxi Prov., Longzhou County; Nonggang Nature Reserve; 29–30 June 2015; Lu Qiu & Qi-Kun Bai leg. • 11 males & 2 females; Yunnan Prov., Yuxi City, Yuanjiang County; 533m; 22 May 2018; Lu Qiu, Wen-Bo Deng & Zhi-Wei Dong leg. Japan • 2 males & 5 females; Okinawa, Mt. Yonahadake; 222m; 10 Aug. 2016; Jian-Yue Qiu & Hao Xu leg.

#### Diagnosis.

**Figure 2.**
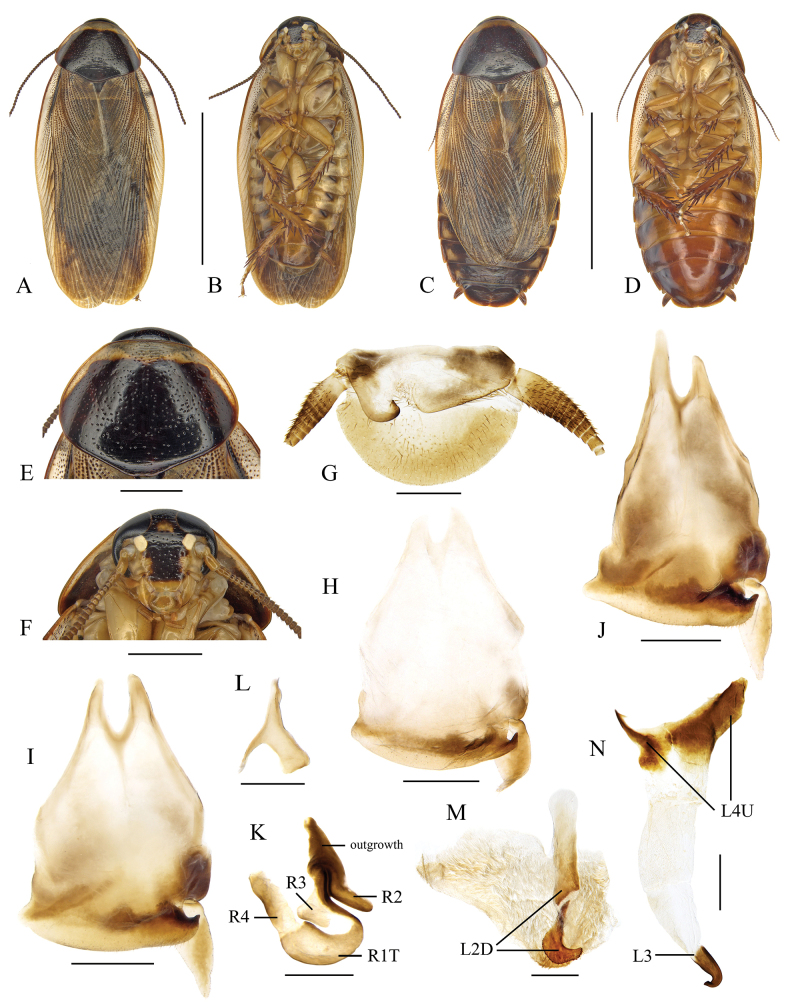
*Pycnoscelusindicus* (Fabricius, 1775) **A, B, E–M** male **C, D** female **A, C** dorsal view **B**, **D** ventral view **E** pronotum, dorsal view **F** head, ventral view **G** supra-anal plate, ventral view **H** subgenital plate, dorsal view (from Yuxi, Yunnan) **I** subgenital plate, dorsal view (from Zimaling, Guangdong) **J** subgenital plate, dorsal view (from Longzhou, Guangxi) **K** right phallomere, dorsal view **L** sclerite R3 of right phallomere, ventral view **M** median phallomere, dorsal view **N** left phallomere, dorsal view. Scale bars: 1.0 cm (**A–D**); 2.0 mm (**E, F**); 1.0 mm (**G–J**); 0.5 mm (**K–N**).

This species is characterized by its pronotum with a broad anterior and narrow anterolateral yellowish band (Fig. 2E), a feature also presents in *P.surinamensis*. The two species are difficult to distinguish in appearance. The key distinction between *P.indicus* and *P.surinamensis* lies in their reproductive modes: *P.indicus* reproduces bisexually, while *P.surinamensis* reproduces parthenogenetically (Roth 1967).

#### Supplementary description of male.

Hind margin of supra-anal plate variable, weakly incised medially, or entire and convexly rounded (Fig. 2G). Sclerotized level and shape of subgenital plate variable: the inner plate nearly symmetrical or strongly asymmetrical, lateral projection on left posterolateral angle distinct or not (Fig. 2H–J). Male genitalia: Right phallomere with caudal part of sclerite R1T widened; R2 slightly curved; the projection arising from the junction of R1T and R2 elongate; R3 forked caudally; R4 plate-like, close to the caudal part of R1; R5 absent (Fig. 2K, L). Sclerite L2D divided into basal and apical parts: the basal part straight; the apical part croissant-shaped with outer margin toothed; apical membrane covered with heavy chaetae (Fig. 2M). Sclerite L3 with hook short and robust, sclerite L4U divided into two parts (Fig. 2N).

#### Measurements (mm).

Body length including tegmen: male 15.8–17.2, female 16.4–18.6; pronotum length × width: male 4.0–4.4 × 5.4–5.9, female 4.3–5.1 × 6.0–6.7; tegmen length: male 13.8–15.3, female 12.7–15.0.

### ﻿ Pycnoscelus nigra

Taxon classificationAnimaliaBlattodeaBlaberidae

(Brunner von Wattenwyl, 1865)

57FF9205-CE93-5FF4-89DA-B55AE590FA9C

Fig. 3

Panchlora nigra Brunner von Wattenwyl, 1865: 280.
Pycnoscelis [sic] nigra: Princis 1964: 274.
Pycnoscelus nigra : Princis 1967: 709; Roth, 1998: 103.

#### Material examined.

China • 1 female; Guangdong Prov., Zhongshan City, Mt. Wuguishan; 5 May 2018; Ke-Liang Wu leg. • 5 males; Yunnan Prov., Xishuangbanna, Mengla County, Menglun Town; 25 May 2016; Lu Qiu & Zhi-Wei Qiu leg. • 1 female; Hainan Prov., Mt. Diaoluo shan; 275 m; 24–25 May 2014; Shun-Hua Gui & Xin-Ran Li leg. • 1 male; Chongqing City, Hechuan District, Dashi Town; 18 July 2013; Zong-Qing Wang leg. • 2 males & 4 females; Sichuan Prov., Panzhihua City, Xinzhuang Village; 1306 m; 15 Oct. 2014; Li He leg. • 6 males; Yunnan Prov., Menglun Town, Xishuangbanna Tropical Botanical Garden, Lvshilin (Green stone forest); 25 May 2016; Lu Qiu & Zhi-Wei Qiu leg. • 1 male; Yunnan Prov., Baoshan City, Mt. Gaoligongshan, Baihualing, Hanlong Zhai; 1400–1900 m; 20–23 June 2020; Lu Qiu & Jin-Lin Liu leg.

#### Diagnosis.

**Figure 3.**
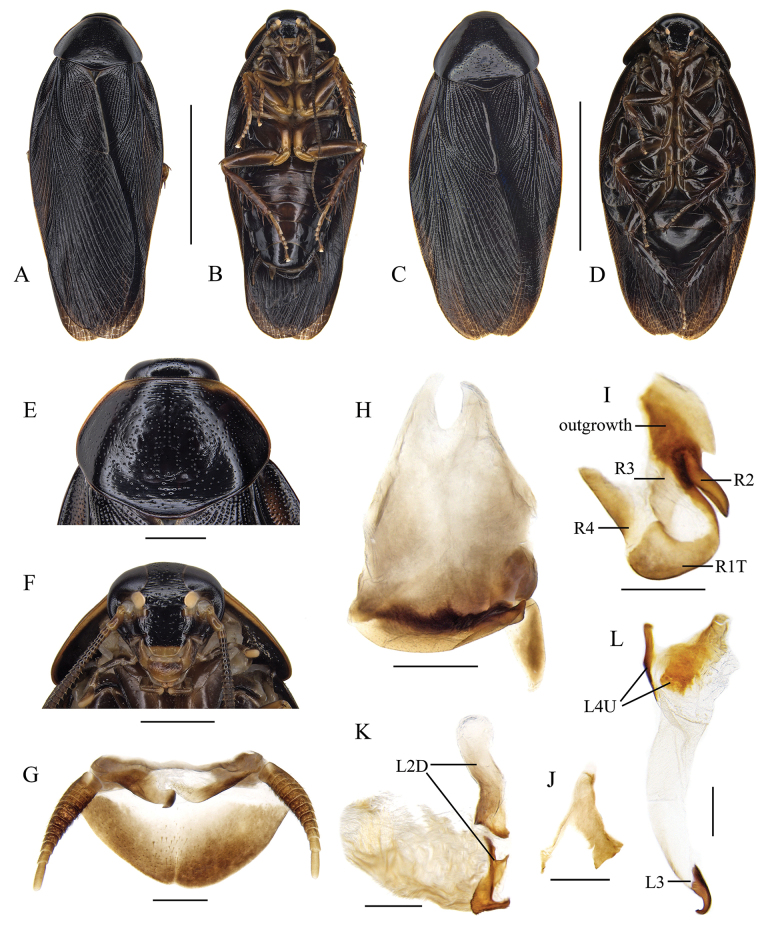
*Pycnoscelusnigra* (Brunner von Wattenwyl, 1865) **A, B, E–K** male **C, D** female **A, C** dorsal view **B**, **D** ventral view **E** pronotum, dorsal view **F** head, ventral view **G** supra-anal plate, ventral view **H** subgenital plate, dorsal view **I** right phallomere, dorsal view **J** sclerite R3 of right phallomere, ventral view **K** median phallomere, dorsal view **L** left phallomere, dorsal view. Scale bars: 1.0 cm (**A–D**); 2.0 mm (**E, F**); 1.0 mm (**G, H**); 0.5 mm (**I–L**).

This species is characterized by the generally dark coloration (Fig. 3A–D) and pronotum with narrow yellow area anterolaterally (Fig. 3E).

#### Supplementary description of male.

Male genitalia: Right phallomere with caudal part of sclerite R1T widened distinctly; R2 nearly straight; the outgrowth arising from the junction of R1T and R2 broad, plate-like; R3 forked caudally; R4 plate-like; R5 absent (Fig. 3I, J). Sclerite L2D with the apical part bifurcated basally; apical membrane covered with heavy chaetae (Fig. 3K). Sclerite L3 with hook comparatively slender, sclerite L4U divided into two parts (Fig. 3L).

#### Measurements (mm).

Body length including tegmen: male 19.5–23.8, female 20.3–24.1; pronotum length × width: male 4.5–4.9 × 5.4–6.9, female 5.0–6.0 × 7.5–8.1; tegmen length: male 15.9–19.6, female 16.5–20.8.

#### Remarks.

Roth (1998) recorded a male of *P.nigra* from Jingdong (= Kintung), Yunnan, and found its left style present. However, the left style is absent in all our new material from Yunnan (Fig. 3H).

### ﻿ Pycnoscelus puteus

Taxon classificationAnimaliaBlattodeaBlaberidae

Wang & Che sp. nov.

76574D9C-1F14-5BFD-B167-61FD879DCEA0

https://zoobank.org/D2D97057-9C9B-40AD-8DFD-63B12C1EB69C

Fig. 4

#### Type material.

***Holotype*.** China • male; Yunnan Prov., Xishuangbanna, Mengla County, Menglun Town, Xishuangbanna Tropical Botanical Garden; 25 May 2016; Lu Qiu & Zhi-Wei Qiu leg.; SWU-B-BB120101. ***Paratypes*.** China • 2 males; same collection data as holotype; SWU-B-BB120102 and 120103 • 1 male; same collection data as holotype; 27 May 2016; SWU-B-BB120104 • 4 males; Yunnan Prov., Pu’er City, Meizihu Lake; Lu Qiu & Zhi-Wei Qiu leg.; 20–21 May 2016; SWU-B-BB120105 to 120109 • 1 male; Yunnan Prov., Xishuangbanna, Jinghong City, Dadugang; 5 May 2013; Zong-Qing Wang leg.; SWU-B-BB120110.

#### Differential diagnosis.

**Figure 4.**
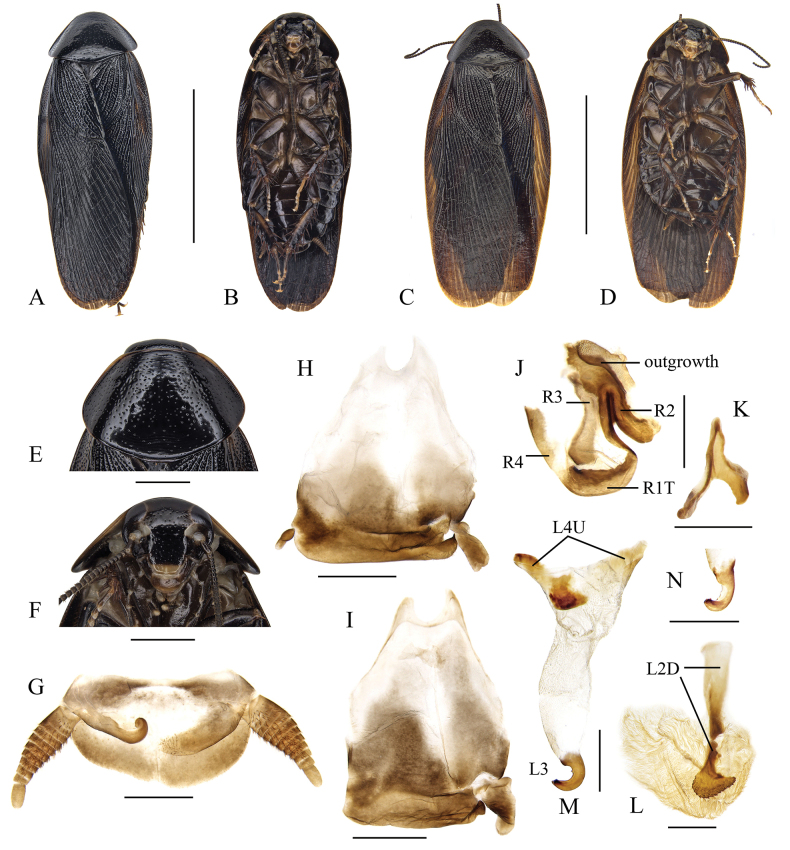
*Pycnoscelusputeus* sp. nov., male **A, B, E–H, J–L** holotype SWU-B-BB120101 **C, D, I, M** paratype SWU-B-BB120105 **A** dorsal view **B** ventral view **C** dorsal view **D** ventral view **E** pronotum, dorsal view **F** head, ventral view **G** supra-anal plate, ventral view **H** subgenital plate, dorsal view (from Xishuangbanna, Yunnan) **I** subgenital plate, dorsal view (from Pu’er, Yunnan) **J** right phallomere, dorsal view **K** sclerite R3 of right phallomere, ventral view **L** median phallomere, dorsal view **M** left phallomere, dorsal view (from Xishuangbanna, Yunnan) **N** left phallomere, lateral view (from Pu’er, Yunnan). Scale bars: 1.0 cm (**A–D**); 2.0 mm (**E, F**); 1.0 mm (**G-I**); 0.5 mm (**J–N**).

*Pycnoscelusputeus* sp. nov. is very similar to *P.nigra* in general appearance and coloration but differs strongly from the later in interocular distance and male genitalia, especially the sclerotized part associated with sclerite R1T and R2 (compare Fig. 3F, I and Fig. 4F, J in present paper). Furthermore, the male genitalia distinguish this new species from all other known species of genus *Pycnoscelus* except *P.rothi*, but the coloration of the pronotum could separate them easily (compare Fig. 4E with Anisyutkin 2002: fig. 1).

#### Description.

**Male (*holotype*).** General color black (Fig. 4A, B). Head black except for yellowish brown clypeo-labral area; maxillary palps and antennae brownish black; eyes black, ocellar spots whitish. Pronotum shining black, with two indistinct, narrow yellow borders anterolaterally (Fig. 4E). Tegmina similar to those of *P.nigra*, dark when folded. Abdomen and legs brownish black. Cerci slightly lighter.

Body slender. Head slightly exposed. Eyes comparatively small; outer margin of ocelli obscure; interocular space wider than the distance between the inner margin of ocellar spots, and smaller than distance between antennal sockets (Fig. 4F). Pronotum subpentagonal, densely punctured (Fig. 4E). Tegmina and wings extending beyond the end of abdomen. Front femur Type C_1_. Hind metatarsus distinctly longer than other segments combined; four proximal tarsomeres with well-developed pulvilli, the one on the first tarsomere occupying almost the complete length of the segment; claws symmetrical and simple; arolium large. Supra-anal plate transverse, slightly asymmetrical, hind margin with a small medial incision (Fig. 4G). Paraprocts of blaberid type, asymmetrical. Subgenital plate asymmetrical, with the right posterolateral corner acute and upturned. Left style minute, right one large and robust (Fig. 4H).

***Male genitalia*.** Right phallomere with caudal part of sclerite R1T rounded; R2 curved; an outgrowth arising from the junction of R1T and R2, with an additional heavily sclerotized part whose surface is pitted; R3 forked caudally, R4 plate-like, R5 absent (Fig. 4J, K). Sclerite L2D divided into basal and apical parts: the basal part short, apically widened; the apical part strongly sclerotized with posterior margin distinctly toothed; apical membrane well developed, with surface covered by microtrichia (Fig. 4L). Sclerite L3 hook with apical incision, inner curved margin with a small tooth; sclerite L4U present and divided into two parts (Fig. 4M, N).

#### Variation.

Body broader, brownish black (Fig. 4C, D); some individuals lack left style (probably missing, Fig. 4I); inner curved margin of hook of sclerite L3 with two small projections (Fig. 4N).

#### Measurements (mm).

Male, body length including tegmina: 20.1–20.9; pronotum length × width: 3.7–4.0 × 5.0–5.6; tegmen length: 16.1–18.0.

#### Etymology.

Derived from the Latin word *puteus*, referring to the pitted surface of the sclerotized enlargement associated with sclerites R1T and R2.

### ﻿ Pycnoscelus undulatus

Taxon classificationAnimaliaBlattodeaBlaberidae

Wang & Che sp. nov.

152CF021-4C72-54AA-9B34-F2FBFD025818

https://zoobank.org/ECA45FFD-3DAE-4200-9FB3-5E2714DC7AEB

Fig. 5

#### Type material.

***Holotype*.** China • male; Guangdong Prov., Guangzhou City, Longyandong Forest Park; 28 June 2015; Zhi-Wei Qiu & Yong-Quan Zhao leg.; SWU-B-BB120201. ***Paratypes*.** China • 1 male; same collection data as holotype; SWU-B-BB120202 • 1 male; Guangxi Prov., Hepu County, Shiwan Town; 14 May 2016; Yi-Zhou Liu leg.; SWU-B-BB120203 • 2 females & 1 nymph; Guangxi Prov., Hepu County, Shiwan Town; 14 May 2016; Yi-Zhou Liu leg.; SWU-B-BB120204 to 120206.

#### Differential diagnosis.

**Figure 5.**
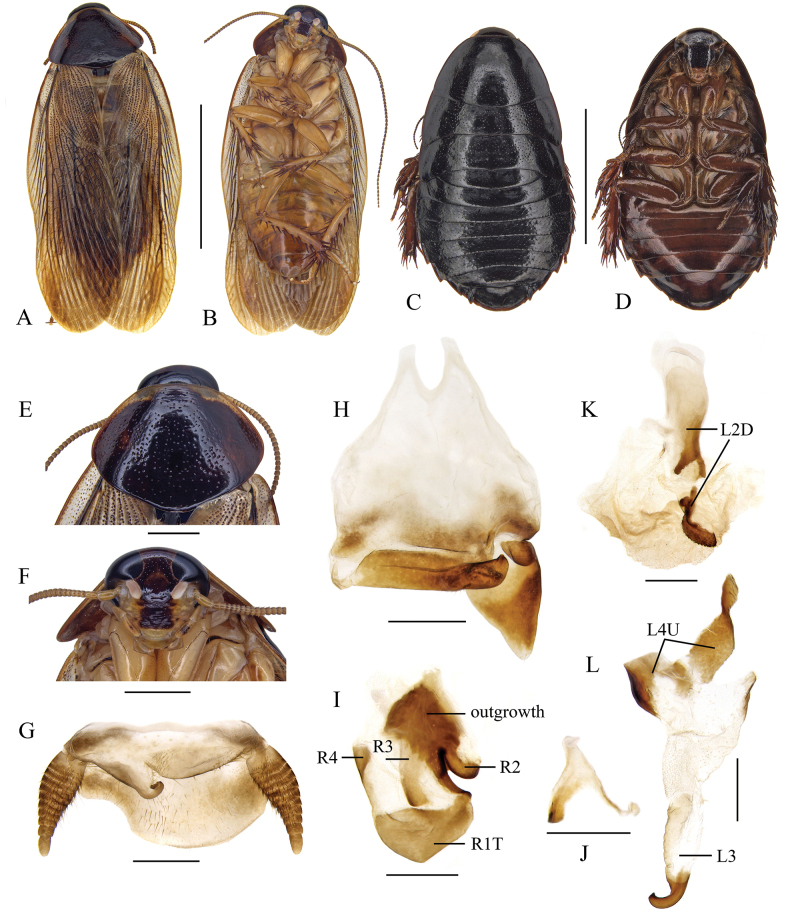
*Pycnoscelusundulatus* sp. nov. **A, B, E–K** holotype, male SWU-B-BB120201 **C, D** paratype, female SWU-B-BB120204 **A** dorsal view **B** ventral view **C** dorsal view **D** ventral view **E** pronotum, dorsal view **F** head, ventral view **G** supra-anal plate, ventral view **H** subgenital plate, dorsal view **I** right phallomere, dorsal view **J** sclerite R3 of right phallomere, ventral view **K** median phallomere, dorsal view **L** left phallomere, dorsal view. Scale bars: 1.0 cm (**A-D**); 2.0 mm (**E, F**); 1.0 mm (**G, H**); 0.5 mm (**I–L**).

The male of this species is similar to *P.vietnamensis* Anisyutkin, 2002 and *P.gorochovi* Anisyutkin, 2002 in the shape of supra-anal plate, which has a deep emargination at the right posterolateral side, but *P.undulatus* sp. nov. differs strongly from these two species by its dark reddish brown pronotum, light marking on facial part between eyes, and the bulky right style (compare Fig. 5H with Anisyutkin 2002: figs 15, 16).

#### Description.

**Male (*holotype*).** General color brownish yellow (Fig. 5A, B). Facial part of head dark reddish brown, with an indistinct three-radial stripe between eyes; other part of head, clypeus, labrum and antennae brownish yellow; maxillary palps brownish yellow with reddish brown maculae; eyes black, ocellar spots whitish. Pronotum reddish brown, with two yellow areas anterolaterally (Fig. 5E). Tegmina brownish yellow. Abdomen yellowish brown to reddish brown. Legs and cerci yellowish brown.

Head slightly exposed. Ocelli subrectangular; interocular space distinctly less than the distance between ocellar spots and antennal sockets (Fig. 5F). Pronotum subpentagonal, densely punctured. Tegmina and wings fully developed, exceeding the end of abdomen. Front femur Type C_1_. Hind metatarsus not quite as long as other segments combined; four proximal tarsomeres with well-developed pulvilli, the one on the first tarsomere occupying almost the whole length of the segment; claws symmetrical and simple; arolium large. Supra-anal plate asymmetrical with hind margin deeply concave at the right side. Paraprocts of blaberid type, asymmetrical. Cerci stout at base (Fig. 5G). Subgenital plate asymmetrical, with the right posterolateral corner slightly obtuse and upturned. Left style absent, right style broadly trigonal (Fig. 5H).

***Male genitalia*.** Right phallomere with caudal part of sclerite R1T irregular and widened; R2 short and curved; a well-developed projection arising from the junction of R1T and R2; R3 forked caudally, R4 plate-like, R5 absent (Fig. 5I, J). Sclerite L2D divided into basal and apical parts: the basal part forked caudally with the inner margin indented; the apical part well sclerotized and more uniform in width than in other *Pycnoscelus* species, posterior margin toothed; apical membrane covered with microtrichia (Fig. 5K). Sclerite L3 hook without an apical incision, inner curved margin with a sharp convexity at apex; sclerite L4U present and divided into two parts (Fig. 5L).

**Females.** Thoracic segments and abdominal tergum black with minute punctures, abdominal sterna and legs reddish brown. Tegmina and wings absent.

#### Measurements (mm).

Male, body length including tegmen: 22.2–23.0; pronotum length × width: 4.3–4.5 × 6.3–7.1; tegmen length: 17.0–20.1. Female, body length: 16.1–20.6; pronotum length × width: 4.35–4.44 × 7.36–7.37.

#### Etymology.

The species epithet is derived from the Latin word *undulatus*, which refers to the caudal edge of basal part of sclerite L2D undulated.

### ﻿ Pycnoscelus semivitreus

Taxon classificationAnimaliaBlattodeaBlaberidae

Princis, 1967

78CD0972-3BF8-55BE-9D18-54EC0DF5219F

Fig. 6

Pycnoscelus semivitreus Princis, 1967: 148; Princis 1971: 1141; Roth 1998: 112.

#### Material examined.

Malaysia • 1 male; Borneo, Mt. Trus Madi, Jungle Girl Camp; 2–5 Oct. 2015; Ye-Jie Lin leg. • 1 male; Borneo, Mt. Trus Madi, Jungle Girl Camp; 3 May 2023; Cai-Xia Yuan leg.

#### Diagnosis.

**Figure 6.**
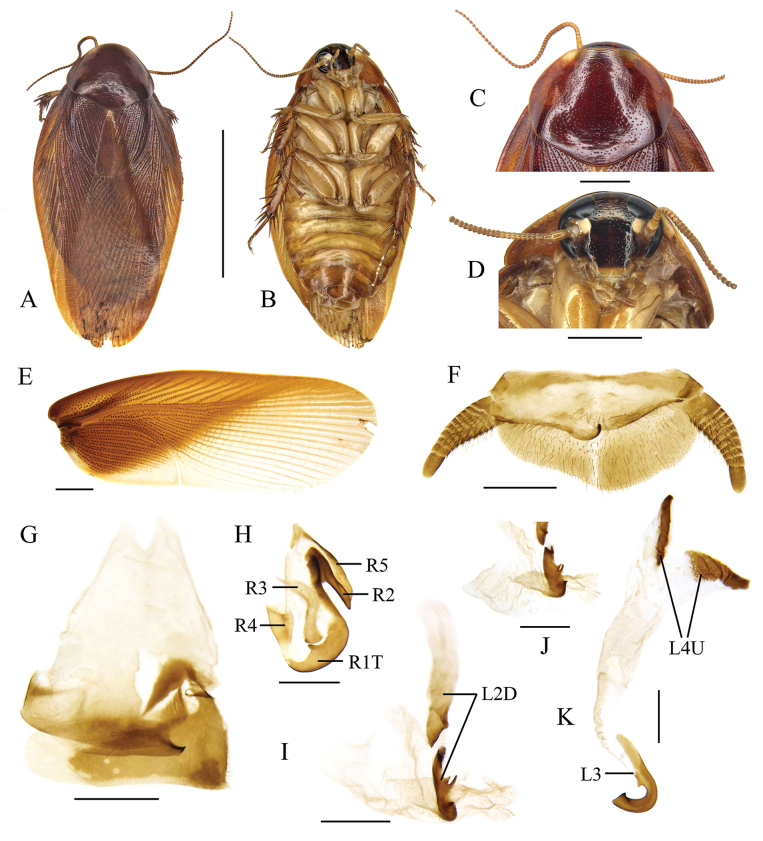
*Pycnoscelussemivitreus* Princis, 1967, male **A** dorsal view **B** ventral view **C** pronotum, dorsal view **D** head, ventral view **E** tegmen, dorsal view **F** supra-anal plate, ventral view **G** subgenital plate, dorsal view **H** right phallomere, dorsal view **I** median phallomere, dorsal view **J** median phallomere, lateral view **K** left phallomere, dorsal view. Scale bars: 1.0 cm (**A, B**); 2.0 mm (**C–E**); 1.0 mm (**F, G**); 0.5 mm (**H–K**).

This species can be easily distinguished from all its congeners by its huge right style, which extends to the left posterolateral angle of subgenital plate (Fig. 6G).

#### Supplementary description of male.

Male genitalia: right phallomere with caudal part of sclerite R1T irregular, inner margin produced at middle; R2 nearly straight, R3 forked, R4 plate-like; R5 elongated, lying above R2 (Fig. 6H). Apical part of sclerite L2D elongated, the right side with several sharp teeth; apical membrane well developed, covered with microtrichia (Fig. 6I, J). Sclerite L3 hook subquadrate apically, inner margin with a tooth at apex; sclerite L4U divided into two parts (Fig. 6K).

#### Measurements (mm).

Body length including tegmen: 20.7; pronotum length × width: 4.7 × 6.2; tegmen length: 17.0.

### ﻿ Pycnoscelus aurantius

Taxon classificationAnimaliaBlattodeaBlaberidae

Hanitsch, 1935

87368253-DA52-5898-8EB5-630AA1095A2A

Fig. 7

Pycnoscelus aurantius Hanitsch, 1935: 18; Bruijning 1947: 210; Roth 1998: 125.
Pycnoscelis [sic] *aurantia*: Princis 1964: 275.

#### Material examined.

Malaysia • 1 male; Sabah, Mt. Trus Madi; 1121m; 5 Oct. 2015; Gui-Qiang Huang leg.

#### Diagnosis.

General color dark orange, similar to *P.semivitreus*, but *P.aurantius* can be readily distinguished from *P.semivitreus* by its smaller right style and the apical part of sclerite L2D blunt without any process.

#### Supplementary description of male.

**Figure 7.**
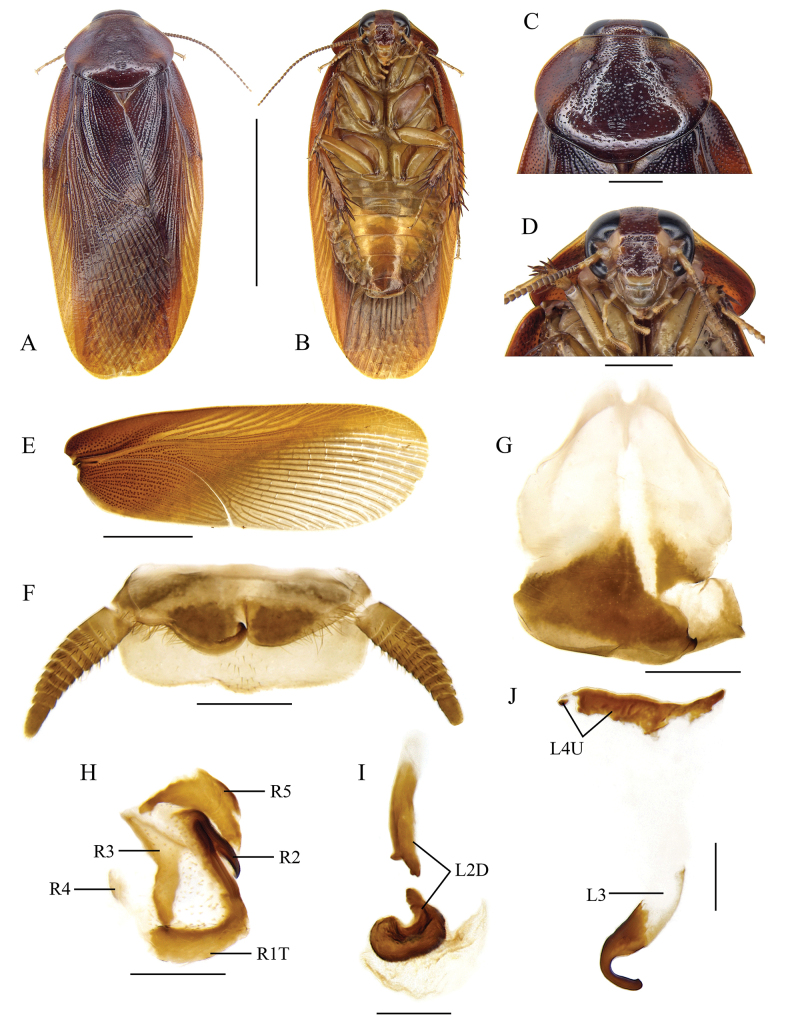
*Pycnoscelusaurantius* Hanitsch, 1935, male **A** dorsal view **B** ventral view **C** pronotum, dorsal view **D** head, ventral view **E** tegmen, dorsal view **F** supra-anal plate, ventral view **G** subgenital plate, dorsal view **H** right phallomere, dorsal view **I** median phallomere, dorsal view **J** left phallomere, dorsal view. Scale bars: 1.0 cm (**A, B**); 2.0 mm (**C, D**); 5.0 mm (**E**); 1.0 mm (**F, G**); 0.5 mm (**H–J**).

Tegmina is somewhat bicolored as *P.semivitreus*, from proximally orange to distally yellowish, with the boundary indistinct (Fig. 7E). Male genitalia: right phallomere with distinct bristles, caudal part of sclerite R1T nearly rectangular, R2 curved, R3 forked, R4 small; R5 lies above R2, irregular, prolonged (Fig. 7H). The basal part of sclerite L2D strongly sclerotized with apical portion less sclerotized and nearly transparent; the apical part of sclerite L2D C-shaped, outer margin without any tooth; apical membrane less developed, covered with microtrichia (Fig. 7I). Sclerite L3 hook slender, inner curved margin with a tooth at apex; sclerite L4U divided into two parts (Fig. 7J).

#### Measurements (mm).

Body length including tegmen: 21.9; pronotum length × width: 4.6 × 6.5; tegmen length: 19.8.

#### Remarks.

The specimen we examined is generally identical to the description of *P.aurantius* provided by Roth (1998). However, Roth (1998) examined the type of *P.aurantius* and found its left style present, whereas this structure is absent in the specimen we examined (Fig. 7G). As only a single specimen is available in our study, it is difficult to determine whether the style is missing or naturally absent. More specimens will be needed in the future to clarify this question, and for now, we have tentatively identified the specimen as *P.aurantius*.

### ﻿ Pycnoscelus striatus

Taxon classificationAnimaliaBlattodeaBlaberidae

(Kirby, 1903)

CFD40A95-E19B-54B3-AF11-5671D88A9CD2

Fig. 8

Leucophaea striata Kirby, 1903: 378; Kirby 1904: 151; Hanitsch 1915: 122; Chopard 1919: 358.
Pycnoscelis [sic] striata: Princis 1964: 274.
Pycnoscelus striatus : Roth, 1998: 117; Lucañas and Lit 2016: 7; Anisyutkin 2018: 83.

#### Material examined.

Malaysia • 5 males & 1 female; Borneo, Sandakan, Gomantong cave; 19 Apr. 2024; Wei-Wei Zhang leg. • 1 male; Borneo, Mt. Trus Madi, Jungle Girl Camp; 19–25 July 2016; Ren-Zhi Zhang leg. • 1 male; Borneo, Mt. Trus Madi, Jungle Girl Camp; 4 May 2023; Cai-Xia Yuan leg.

#### Diagnosis.

This species is close to *P.semivitreus* in the structure of male genitalia, but the shape of the right style could distinguish them easily: extending over the middle of subgenital plate in *P.striatus* (vs more elongate and extending to the left posterolateral angle of subgenital plate in *P.semivitreus*).

**Figure 8.**
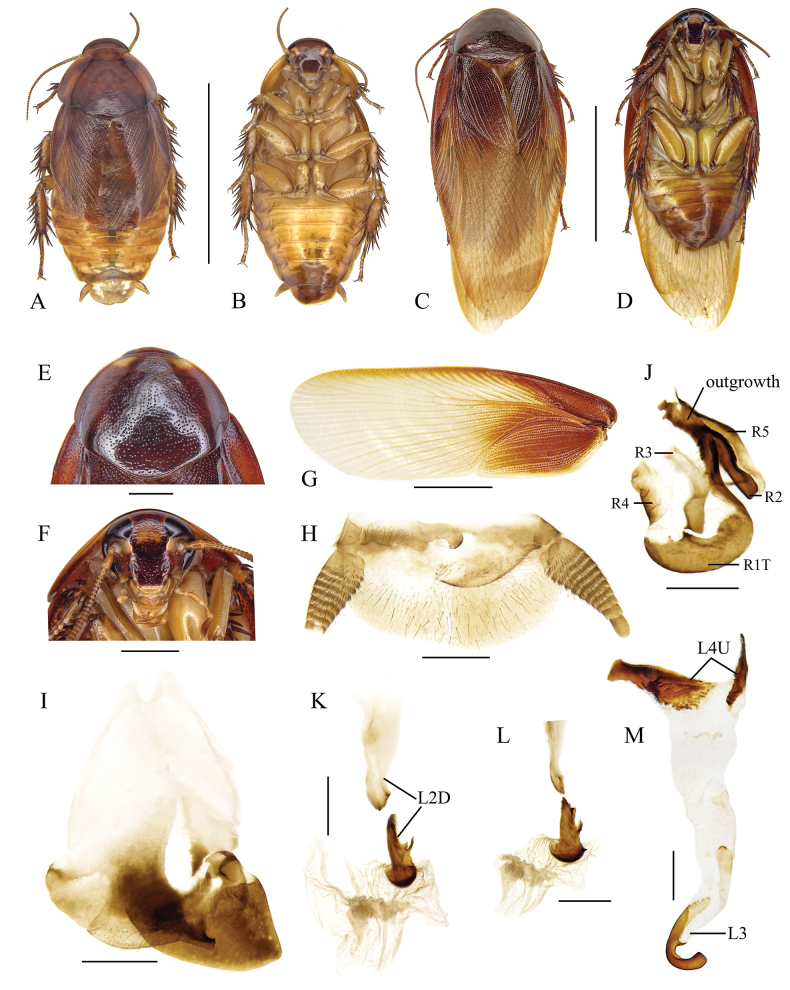
*Pycnoscelusstriatus* (Kirby, 1903), male **A, B** brachypterous **C, D** macropterous **A, C** dorsal view **B, D** ventral view **E** pronotum, dorsal view **F** head, ventral view **G** tegmen, dorsal view **H** supra-anal plate, ventral view **I** subgenital plate, dorsal view **J** right phallomere, dorsal view **K** median phallomere, dorsal view **L** median phallomere, lateral view **M** left phallomere, dorsal view. Scale bars: 1.0 cm (**A–D**); 2.0 mm (**E, F**); 5.0 mm (**G**); 1.0 mm (**H, I**); 0.5 mm (**J–M**).

#### Measurements (mm).

Macropterous, male, body length including tegmen: 23.6; pronotum length × width: 5.7 × 7.0; tegmen length: 20.8. Brachypterous, body length (from vertex to tip of abdomen): male 14.0–15.0, female 15.0; pronotum length × width: male 3.7–4.1 × 5.4, female 4.2 × 5.6; tegmen length: male 7.4–7.5, female 7.1.

### ﻿ Pycnoscelus

Taxon classificationAnimaliaBlattodeaBlaberidae

sp. E

5727C4BD-D4AE-5216-84CB-D17B06E59B16

Fig. 9

#### Material examined.

Malaysia • 1 male; Borneo, Mt. Trus Madi, Jungle Girl Camp; 2–5 Oct. 2015; Ye-Jie Lin leg. • 1 male; Borneo, Mt. Trus Madi, Jungle Girl Camp; 3 May 2023; Cai-Xia Yuan leg.

#### Diagnosis.

This species can be readily distinguished from other *Pycnoscelus* species by the color pattern of pronotum and tegmina. The apical part of sclerite L2D of this species is also peculiar in this genus (see description below for details).

#### Description.

**Figure 9.**
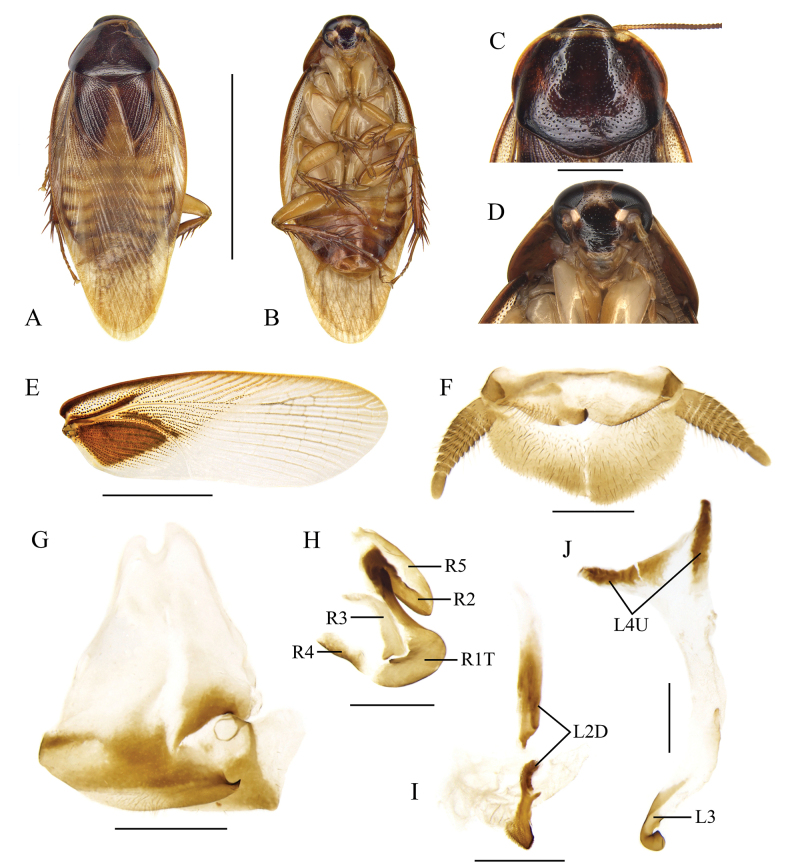
*Pycnoscelus* sp. E, male **A** dorsal view **B** ventral view **C** pronotum, dorsal view **D** head, ventral view **E** tegmen, dorsal view **F** supra-anal plate, ventral view **G** subgenital plate, dorsal view **H** right phallomere, dorsal view **I** median phallomere, dorsal view **J** left phallomere, dorsal view. Scale bars: 1.0 cm (**A, B**); 2.0 mm (**C, D**); 5.0 mm (**E**); 1.0 mm (**F, G**); 0.5 mm (**H–K**).

General color yellowish brown, with pronotum, tegmina, and abdominal sternites partly reddish brown (Fig. 9A, B). Facial part of head dark brownish, with a three-radial stripe between eyes; other part of head, clypeus, labrum and antennae yellowish brown; eyes black, ocellar spots yellow white. Pronotum dark reddish brown, with a yellow stripe along the anterior margin (Fig. 9C). Tegmina bicolored, the dark part, principally half anal field reddish brown, remainder yellowish hyaline; the boundary between the two regions extends obliquely from the left posterolateral side of the anal field to half of the radial field; in the dark region, dark brown spots scattered along veins (Fig. 9E). Abdomen yellowish, with last sternites reddish brown. Legs yellowish brown except for reddish brown tibia and tarsomere. Cerci reddish brown.

Head slightly exposed. Interocular space narrower than the distance between ocellar spots and antennal sockets (Fig. 9D). Pronotum subpentagonal, densely punctured. Tegmina and wings fully developed, exceeding the end of abdomen. Front femur Type C_1_. Hind metatarsus slightly longer than other segments combined; four proximal tarsomeres with well-developed pulvilli, the one on the first tarsomere occupying almost the whole length of the segment; claws symmetrical and simple; arolium large. Supra-anal plate weakly asymmetrical, covered with bristles; paraprocts of blaberid type, asymmetrical (Fig. 9F). Subgenital plate asymmetrical, with the right posterolateral corner sharp and upturned. Left style absent, right style plate-like, broadly trigonal (Fig. 9H).

***Male genitalia*.** Right phallomere with caudal part of sclerite R1T rounded, inner margin produced apically; R2 nearly straight; R3 forked caudally, with left branch projected at apex; R4 plate-like; R5 lies above R2 and fused with its distal part (Fig. 9H). Sclerite L2D divided into basal and apical parts, the apical part well sclerotized, irregular, with distal part hairy instead of toothed; apical membrane less developed (Fig. 9I). Sclerite L3 hook very short, with the apex widened; sclerite L4U present and divided into two parts (Fig. 9J).

#### Measurements (mm).

Body length including tegmen: 17.7–18.0; pronotum length × width: 3.8–4.0 × 4.4–4.8; tegmen length: 14.7.

#### Remarks.

According to the description of *P.rufus* given by Anisyutkin (2004), *Pycnoscelus* sp. E closely resembles this species in general color pattern, the shape of right style and right phallomere, but differs in features of pronotum and the apical part of sclerite L2D. We refrain from defining *Pycnoscelus* sp. E as a new species until we examine the type specimen of *P.rufus*.

### ﻿ Pycnoscelus

Taxon classificationAnimaliaBlattodeaBlaberidae

sp. F

D0DBC751-4DEB-5DAF-9DC1-293227C5B53A

Fig. 10

#### Material examined.

China • 1 female; Yunnnan Prov., Mengla County, Menglun Town, Manbian Cun; 30 July 2009; Zong-Qing Wang leg.

#### Measurements (mm).

Body length: 17.4; pronotum length × width: 4.3 × 7.8; tegmen length: 2.5.

#### Remarks.

*Pycnoscelus* sp. F is currently represented by a single female specimen. This specimen is characterized by its strongly reduced tegmina (lobiform and lateral) and the complete absence of hind wings. Wing reduction is also observed in the females of *P.micropterus* Hanitsch, 1931, *P.striatus* (Kirby, 1903), *P.femapterus* Roth, 1998, and *P.undulatus* sp. nov. However, *P.micropterus* and *P.striatus* differ from *Pycnoscelus* sp. F by the tegmina reaching the abdominal terga, and the presence of hind wings. Furthermore, *P.femapterus* and *P.undulatus* sp. nov. can be distinguished from *Pycnoscelus* sp. F by the complete lack of both tegmina and wings.

**Figure 10.**
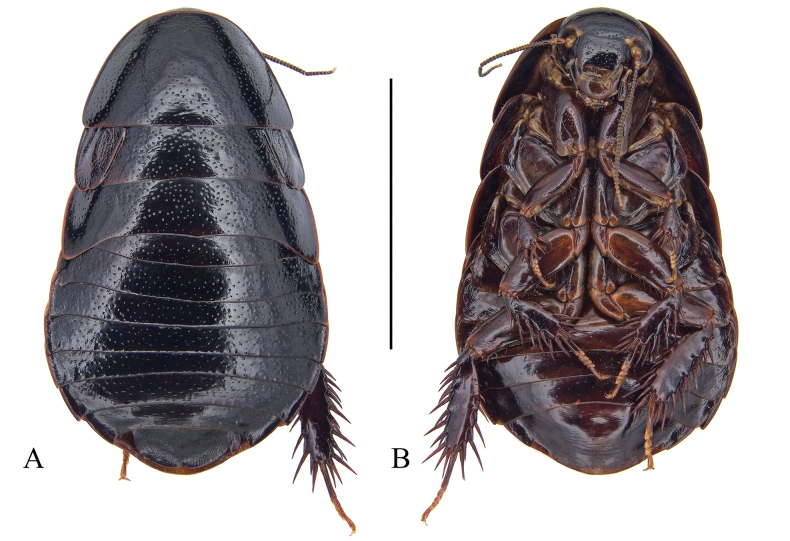
*Pycnoscelus* sp. F, female **A** dorsal view **B** ventral view. Scale bar: 1.0 cm.

It is worth noting that no female specimens of *P.puteus* sp. nov. have been recorded, and both *P.puteus* sp. nov. and *Pycnoscelus* sp. F were collected from Menglun Town, Mengla County, Yunnan Province. It is plausible that *Pycnoscelus* sp. F represents the female form of *P.puteus* but it needs further study.

## ﻿Discussion

Classifying species into groups is essential for understanding their putative phylogenetic relationships and for rapid identification. Anisyutkin (2003) previously noted that male genitalia structures, particularly sclerite L2D, often show similarities between closely related species; he used this feature as the primary criterion to divide the genus *Rhabdoblatta* into groups. Similarly, Roth (1989) suggested that sclerite L2D could effectively indicate relationships among *Paranauphoeta* species.

For the genus *Pycnoscelus*, Roth (1998) proposed two species groups based solely on the shape of the right style: the *indicus* species group and the *striatus* species group. In this study, we examined eight *Pycnoscelus* species, for which male genitalia characters were obtained. Combining the data from Anisyutkin (2002, 2004, 2018), we found distinct differences between the species of the *indicus* group and those of the *striatus* group (summarized in Table 1). Specifically, species in the *indicus* group, including *P.indicus*, *P.nigra*, *P.puteus* sp. nov., *P.undulatus* sp. nov., *P.gorochovi*, *P.rothi*, *P.vietnamensis*, and *P.schwendingeri*, share the absence of sclerite R5 and the apical part of sclerite L2D with its caudal margin toothed. In contrast, species in the *striatus* group, comprising *P.aurantius*, *P.rufus*, *P.striatus*, and *P.semivitreus*, have sclerite R5 present, and the apical part of sclerite L2D with untoothed caudal margin. These differences in male genitalia characters can be used to further distinguish the two groups. The remaining species, *Pycnoscelus* sp. E, differs from all other species by the feature of sclerite L2D, whose apical part is neither toothed nor smooth but covered with chaetae. Thus, the position of *Pycnoscelus* sp. E within the species groups remains unclear.

**Table 1.** Characters of *Pycnoscelus* species (characters of *P.gorochovi* Anisyutkin, 2002, *P.rothi* Anisyutkin, 2002, *P.vietnamensis* Anisyutkin, 2002, *P.rufus* Bey-Bienko, 1950 and *P.schwendingeri* Anisyutkin, 2018 were gathered from literature). I – right style elongate and cone-shaped (0), broader and plate-like (1); II – apical part of sclerite L2D with caudal margin toothed (0), smooth (1), covered with chaetae (2); III – R5 present (0), absent (1); IV – outgrowth arising from the junction of sclerite R1T and R2 absent (0), present (1).

Species	Characters			
	I	II	III	IV
*P.indicus* (Fabricius, 1775)	0	0	1	1
*P.nigra* (Brunner von Wattenwyl)	0	0	1	1
*P.puteus* sp. nov.	0	0	1	1
*P.undulatus* sp. nov.	0	0	1	1
*P.gorochovi* Anisyutkin, 2002	0	0	1	1
*P.rothi* Anisyutkin, 2002	0	0	1	1
*P.vietnamensis* Anisyutkin, 2002	0	0	1	1
*P.schwendingeri* Anisyutkin, 2018	0	0	1	1
*P.rufus* Bey-Bienko, 1950	1	1	0	0
*P.semivitreus* Princis, 1967	1	1	0	0
*P.aurantius* Hanitsch, 1935	1	1	0	0
*P.striatus* (Kirby, 1903)	1	1	0	1
*Pycnoscelus* sp. E	1	2	0	0

Furthermore, the outgrowth arising from the junction of sclerites R1 and R2 is likely an apomorphy of the genus *Pycnoscelus*, as such a structure is quite rare in other Blaberidae taxa. In this case, the absence of this outgrowth in *P.aurantius*, *P.rufus*, and *P.semivitreus* is possibly a plesiomorphic condition, also suggesting a close relationship beween them. On the other hand, the simultaneous presence of the outgrowth and sclerite R5 in *P.striatus* may represent an autapomorphy within *Pycnoscelus*.

Although we proposed additional male genitalia characters to further distinguish and delineate the two species groups of *Pycnoscelus*, these are based only on species with known male genitalia data; information on the male genitalia of 15 species is still lacking. Describing the male genitalia of these species and acquiring molecular data in future studies would validate the proposed characters and greatly enhance our understanding of the systematics of *Pycnoscelus*, thereby enabling a more comprehensive classification.

## Supplementary Material

XML Treatment for Pycnoscelus

XML Treatment for Pycnoscelus indicus

XML Treatment for Pycnoscelus nigra

XML Treatment for Pycnoscelus puteus

XML Treatment for Pycnoscelus undulatus

XML Treatment for Pycnoscelus semivitreus

XML Treatment for Pycnoscelus aurantius

XML Treatment for Pycnoscelus striatus

XML Treatment for Pycnoscelus

XML Treatment for Pycnoscelus

## ﻿Additional information

### Author ORCIDs

Yi-Shu Wang https://orcid.org/0009-0008-5507-760X

Zong-Qing Wang https://orcid.org/0000-0001-9413-1105

Yan-Li Che https://orcid.org/0000-0003-3214-9494

### Data availability

All of the data that support the findings of this study are available in the main text.
